# The next stage of physiological anthropology

**DOI:** 10.1186/s40101-023-00320-9

**Published:** 2023-03-10

**Authors:** Akira Yasukouchi

**Affiliations:** Fukuoka, Japan

**Keywords:** Physiological anthropology, Functional coordination, Functional potentiality, Physiological adaptation

## Physiological anthropology and human biology

The scope of physiological anthropology is, in part, similar to that of human biology and biological anthropology. Raymond Pearl first proposed the term human biology and founded the journal *Human Biology* in 1929. In the study of human biology, emphasis is placed on the population level, including biometric analyses and experimental studies. The topics covered included population structure and vital statistics, reproduction, growth, aging, longevity, genetics, disease, and nutrition [1]. The *Annals of Human Biology*, founded in 1974, and the *American Journal of Human Biology*, founded in 1989, also emerged within the mainstream of this field.

In physical anthropology, Washburn [2] proposed the idea of “new physical anthropology” in 1951, which deals with topics such as variations in human adaptation, population genetics, natural selection, and evolutionary processes. Within the academic societies for physical anthropology, these studies were also referred to as biological anthropology. The Human Adaptability Project led by Joseph S. Weiner as part of the International Biological Program (IBP) from 1964 to 1974 globally accelerated the study of human biology by promoting standardization of methods and cooperation in multidisciplinary projects. After the IBP studies, Damon published *Physiological Anthropology* in 1975 [3], which is a notable book for Japanese physiological anthropologists. The topics covered included the main physical factors of the environment, such as light, high and low ambient temperature, high altitude, and noise in addition to nutrition, infectious disease, behavior, and ecology, for which the practical existence of intra- and inter-population differences in physiological responses had already been verified. Damon was also greatly concerned with new and complex stresses derived from urbanization. Also, around 1975, the interests of American physiological anthropologists shifted to topics related to health in connection with the outcomes of complex biological, social, and ecological factors, with less emphasis placed on evolutionary processes [4].

In Japan, Masahiko Sato founded the *Annals of Physiological Anthropology* in 1983, which subsequently changed its name to the *Journal of Physiological Anthropology* in 2006. This is the official journal of the Japan Society of Physiological Anthropology, and 2023 will mark its 40th anniversary. Previous eras of physiological anthropology have been reviewed by Sato et al. [5] in 1983 and Sato [6] in 1995, which covered the significant implications of studies of Paul T. Baker, Joseph S. Weiner, Nigel A. Barnicot, and other biological anthropologists, as well as studies of human adaptability in the IBP. A major topic in IBP research was human adaptability to tolerate high and low ambient temperatures and hypoxic environments at locations around the world. Sato and colleagues, on the other hand, had commenced laboratory studies in the early 1970s using sophisticated climate chambers and evaluated physiological adaptability to environments under various combinations of physical factors including temperature, humidity, air flow, air pressure, and light through elaborate physiological measurements. These studies included not only extreme environmental stresses, but also mild to moderate stresses. Using climate chambers, physiological anthropologists evaluated physiological adjustments in a state of acclimation but not in a state of acclimatization. In addition, field studies were also conducted to evaluate the functional adaptability to the living environment and to examine the regional and population differences [7, 8], though the studies were limited to within Japan at that time. Furthermore, since the 1980s, physiological anthropologists have examined mental stresses by employing increasingly sophisticated devices for physiological measurement including electroencephalograph and have also sought to evaluate emotional aspects such as empathy in a community [9, 10] and attachment between mother and child [11].

The conceptual framework of physiological anthropology since 2000 was discussed by Sato [12], who suggested that physiological anthropologists generally begin with physiological and morphological measurements taken with much more accurate equipment and methods than in the past, enabling the evaluation of individual characteristics to elucidate individual differences. Whereas the mean value is simply a statistical abstraction, the variations in physiological responses are the reality. This way of thinking is called individual thinking in physiological anthropology in Japan.

Physiological anthropology and human biology have shared a focus on human adaptability to the living environment and evaluated the relationship between variation and adaptability. However, human biology generally emphasizes human population biology at the global level, while physiological anthropology is interested in physiological adaptability, especially in human-made environments, through individual thinking and variations in adaptive responses at both the individual and population levels.

## General perspectives in physiological anthropology

**Fig. 1:**
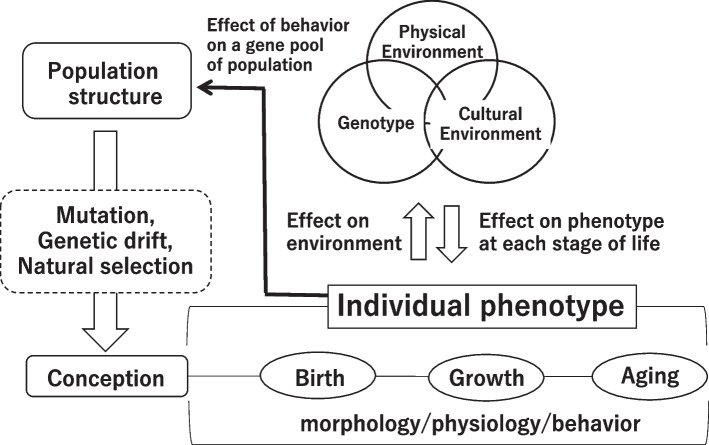
Effects of living environment on phenotype throughout an individual’s lifetime from conception to each stage of birth, growth, and aging

The effects of living environment on phenotype throughout an individual lifetime from conception to each stage of birth, growth, and aging are shown in Fig. 1. Baker [13] proposed the eternal triangle of physical environment, culture, and genotype as factors affecting phenotype. Figure 1 shows these three factors (here called the 3Fs) and the interaction among them to form morphological, physiological, and behavioral phenotypes. Originally, behavior is driven by emotional systems based on rewards and aversion, and thus, behavior is an important consideration given that modern technology allows humans to intentionally change their environment to minimize effort or to feel comfort. This tends to lead to habituation. Habitual behavior constitutes a behavioral history that largely determines long-term environmental stress. On the other hand, individual phenotypes or behaviors collectively affect population structure or the gene pool of the population through things such as migration and mate selection. This in turn modified the genetic history of our ancestors and gave rise to biological phenomena such as mutations, genetic drift, and natural selection, forming the backdrop to the conception of each individual. After conception, the 3Fs affect individual phenotypes during the person’s lifetime while they retain the same DNA. Also, the physical resources available to a fetus or infant would be partly affected by the behavioral history of the mother [14], such as her nutrient intake [15, 16] and time-dependent daily activities forming circadian rhythms [17], for example. Phenotypes during growth would be modified in part by developmental adaptations as proposed by Frisancho [18], which promote fitness in the living environment before reaching adulthood. For example, the residual volume of the lungs, the number of active sweat glands, the ratios of white and brown adipose tissue, and the ratios of fast- and slow-twitch muscle fibers might be shaped primarily after birth as developmental adaptations that might arise from interactions between genes and the environment. The 3Fs and the time factor of aging (or, at a molecular level, changes in DNA methylation) together with behavioral history affect phenotypes in the elderly in part due to epigenomic factors [19]. In general, epigenetics is important to keep in mind when investigating phenotypic adjustments that occur during a lifetime.

Physiological anthropologists are interested in functional responses to stresses encountered in the living environment. These stresses generally depend on an individual’s behavioral history in a given living environment (e.g., habitually avoiding severe stresses or easing stresses by means of modern conveniences). Functional adjustments resulting from physiological and related morphological features should be evaluated in terms of whether they are adaptive. When plastic changes in the efficiency and tolerance of physiological functions contribute to benefits in daily activities and survival, the adjustments can be regarded as adaptive responses.

## A conceptual framework of functional coordination and functional potentiality

The plasticity of physiological adjustments is affected by behavioral history via the physical and cultural environments, and differences in behavioral history would affect the degree of plasticity. The resulting variations in the adjustments would at least consist of differences in functional coordination and functional potentiality. These differences are the focus of physiological anthropologists for evaluating physiological adaptation. The main physiological functions related to survival are work capacity and homeostasis accommodated by the coordination of hierarchical lower-level functions, for example, from the molecular level to respiratory and circulatory functions that shape the cardio-respiratory system. In addition, there is, for example, reciprocal control between the sympathetic and parasympathetic autonomic nervous systems to accommodate particular tissues or organs, as well as motor neuronal and hormonal controls that work together to form an integrated system for the coordination of the whole body. In terms of body temperature regulation, the body responds to heat or cold stress via the coordinated functional system comprising vascular control, heat production, sweating, and other functions. A degree of acclimatization would be determined mainly by the differences in environmental conditions and individual behavioral history inducing variation in functional changes.

When there is an increase in the frequency of severe stress beyond the stress experienced in daily life over a long period of time, acclimation or acclimatization progresses and modifies the related functional system, resulting in a new set of functional patterns enhancing efficiency and stress tolerance. It is likely that when people raised in Japan move to a much colder region such as Alaska and stay there for a long time, newly improved non-shivering heat production adds to the ordinary functional system that is itself modified at the same time, resulting in the formation of a new coordinated pattern. This acclimatization enhances cold tolerance related to survival. There are two main means of adaptive adjustment—one is modifying the ability to tolerate a fatal state under maximal stress (resistance adaptation), and another is modifying functional efficiency against mild or moderate stress (capacity adaptation) [20].

Another important aspect to be considered is functional potentiality, which exists when functional capacity and functional efficiency are reversible with plasticity. Maximal oxygen intake is a limiting factor in physical work capacity but can be increased within genetic/epigenetic/limits by repeated physical training and decreases again when physical training is stopped. As for functional efficiency, physical exercise increases cardiac output by increasing the product of stroke volume and heart rate in a coordinated response. Increased cardiac output is determined partly by the increase in heart rate and partly by the increase in stroke volume under mild to moderate loads, while increased heart rate is the dominant factor, with stroke volume almost a maximum, under moderate to maximal load. During physical exercise with a gradually increasing load, better functional efficiency is kept when cardiac output is increased through increases in both stroke volume and heart rate than through an increase in heart rate alone. In other words, efficiency is higher during mild to moderate exercise than during moderate to severe exercise. The critical load at which the increase of heart rate becomes dominant is increased by repeated physical training. A new combination of stroke volume and heart rate with higher efficiency at higher physical load shows improvement in cardiovascular tolerance or maximal oxygen intake. Such adjustments can be regarded as an adaptive response in terms of physical fitness. This could be caused by the new manifestation of a potential function, in other words, the activation of reserved capacity.

In general, a degree of potentiality also depends on the magnitude, frequency, and duration of stresses induced by environmental conditions and related behavioral history. If biologically innate maximal capacity was more or less constant, this would imply that manifested functions and potential functions would be two sides of the same coin [21–24].

## Evaluation of physiological adaptability to the modern environment

It is said that human biological adaptability was established during the hunter-gatherer age of the Paleolithic, which occupied more than 95% of our existence, but we now live in a much different environment. Nevertheless, our biological adaptability is fundamentally the same now as it was in the past.

Physiological anthropologists are deeply interested in the evaluation of functional responses to the modern environment created by highly advanced science and technology that our ancestors never experienced. The discrepancy between the two environments pushes us forward to reevaluate the relationship between functional adjustments to current stress and adaptability to it. In humans, upright posture and bipedalism are adaptations enabling long-distance hunts, but many contemporary humans do sedentary office work all day, which tends to cause low back pain and decreased skeletal muscle mass and strength. We are likely to use air conditioning to maintain thermal comfort throughout the day, regardless of the season or our geographical location, which might weaken heat and cold tolerances. Also, artificial lighting can illuminate our entire living environment, even deep into the night if we like, thereby affecting our biological rhythms and leading to problems such as delayed sleep phase disorder and insufficient sleep. A physiological basis for this is called non-image-forming responses to light [25], which might induce sleep disorder, depression, and other diseases [26, 27]. Regarding the effects of behavioral history in daily life, Maeda et al. [28] reported that people who tend to eat between meals exhibit a lower basal metabolic rate (BMR) and that people with lower physical activity also exhibit a lower BMR. People with a lower BMR due to such a behavioral history show lower tolerance to cold stress [29].

Small but long-lasting stresses, or invisible stresses coming from the modern environment, were less common in the past and significantly affect intrinsic functional adjustments. This means that the invisible stresses might not only weaken functional adaptability (or enlarge functional potentiality) but also cause disharmony due to the adjustments, such as a delayed circadian rhythm phase. With many people living a modern lifestyle, the range of variations is becoming smaller with fewer physical stresses, and the biological meaning of variations has to be reconsidered with special reference to mismatched stresses such as lighting at night and the use of air conditioning across all seasons. Such invisible stresses have seldom been observed before to evaluate physiological adaptability. For this reason, physiological anthropologists also focus on individual behavioral history because a person intentionally chooses modern conveniences and artificially changes the living environment independent of natural conditions.

In light of the above, the Japan Society of Physiological Anthropology (JSPA) has stated that “Rapid advances in science and technology are having a profound effect on the human community, in terms of not only lifestyle and culture but the physiological capabilities of the human body as well.” cited by the website of JSPA.

## Applied studies of physiological anthropology

One feature of physiological anthropology is joint research with the manufacturing industry from the early 1990s. As described above, studies in this field have investigated functional adaptability to a modern living environment, particularly identifying the effects of invisible or unperceived stresses. The outcomes of these studies should be put into practical use. In the 1990s, some industries fortunately became aware and suspected that innovative technological advances might cause some kinds of problems in human beings. For industries that provide many kinds of products that are brought into a living environment, joint research is a useful way to contribute to the creation of a human-centric environment. Such applied studies have investigated factors including temperature, light, sound [30], odors [31], and textures of materials [32–34]. There are studies not only on negative effects but also on positive effects such as the ability to recover from problems and to be comfortable without invisible stresses [35, 36].

Many applied studies have been conducted in collaboration with industry in regard to thermal conditions [37] and lighting conditions [38]. The following presents an example of a study on lighting conditions with home appliances. Lighting conditions are an important factor in the office environment because the illuminance and color temperature (light color) of light sources and the timing and duration of light exposure affect arousal level, autonomic nervous system tone, hormonal secretions, the immune system, and biological rhythms through the retinohypothalamic tract [39] and then affect productivity as well. Recently, almost all light sources have been replaced with light-emitting diodes, enabling precise control of illuminance levels and color temperature throughout the day. In joint research with industry, it was demonstrated that conditions of higher illuminance and higher (cooler) color temperature in the morning and lower illuminance and lower (warmer) color temperature from 1 h after lunch would better maintain circadian rhythm and arousal level as well as productivity during the daytime in comparison with constant lighting conditions. This was demonstrated through measurements of event-related potential, heart rate variability, melatonin secretion, rectal temperature, and reaction time [40]. This type of lighting system has now been implemented into living environments for practical use [41].

Recent applied studies have been conducted not only for physio-anthropological evaluation of products themselves but also to search for and create new research themes that will be relevant to society in the near future.

## The next stage of physiological anthropology

Experiments in the laboratory were necessary for elucidating functional efficiency and tolerance through accurate physiological measurements. Physiological anthropologists have been acutely aware that experimental studies alone are not enough to evaluate realistic functional adaptability in humans because of small sample sizes with participants mostly limited to healthy young adults. The experimental conditions are also controlled in a climate chamber with only a single factor changed while the other variables are kept constant, which is much different from real-world conditions. Therefore, the results of experimental studies need to be verified in field studies. Recently, some work has been done to link field studies to laboratory results [42, 43].

In the next stage of physiological anthropology, field studies linked with experimental results should be carried out with three objectives in mind. The first is the verification of experimental results. The second is the investigation of how the plasticity of functional adjustments is derived from interactions between genes and the environment, as well as the investigation of the mechanism of predicted adaptations, developmental adaptations, and decline due to aging. Rapid changes in the modern environment due to technological and cultural changes occurring rapidly, even within a generation, will affect the biological aspects of fetuses and infants including their body growth, for example. Recently, Japanese women have experienced social pressure to diet in order to stay slim during pregnancy, which might affect the metabolic systems of the fetus [44]. Meanwhile, extended use of air conditioning during the summer or in tropical regions might affect the number of active sweat glands in infants. Children nowadays tend to stay indoors and have less exposure to sunlight, causing higher sensitivity to light, so lighting at night might exacerbate the effects on their circadian rhythm, sleep, and other non-image-forming responses, and these effects might be much stronger in children than in adults because of the higher light transmittance of the crystalline lens during childhood [45]. These examples show that we must study how rapid changes in technology and culture within a generation affect children and their growth through predicted and developmental adaptations. Also today, people have greater longevity than ever before, and advances in technology will also greatly affect the lifestyle of the elderly. Going forward, physiological anthropologists should examine the validity of innate functional adaptability to the environment as well as individual and population differences from the perspective of epigenetics and seek to elucidate environmental effects and their changes over time as seen in secular trends.

To date, physiological anthropologists have put less emphasis on population adaptation. The third area where field studies are needed is the relationship between functional adjustments and mental and physical stresses in a given environment and genotype, approached from the perspective of evaluating population adaptation. Such physio-anthropological studies would be challenging, but work is now underway.

In 2013, Higuchi et al. [46] found that the pupillary light reflex associated with melanopsin gene polymorphisms exhibited a significant interaction between the 1394T genotype (TT versus TC+CC) and pupil size according to illuminance level. Pupil size was significantly smaller in participants with the C allele than in those with the TT genotype at a higher illuminance level. It was also found that the C allele frequency of 1394T was greater in the European population than in the Asian population, including Japan, according to the database of the international HapMap Project, suggesting that one cause might be that the European population has higher intraocular stray light due to lighter pigmentation of the iris [47]. According to a study by Akiyama et al. [48] in 2017, one of the three haplotypes of the PER2 clock gene was significantly associated with sensitivity to light-induced melatonin suppression at night. They speculated that low sensitivity was the ancestral type and that relatively higher sensitivity has spread worldwide since the early human migration out of Africa, as judged from data on global haplotype frequencies. These data are not enough to verify functional adaptation at the population level and evolutionary significance. Further studies are now ongoing to address functional adjustment to the daily environment in association with genotype.

The following three objectives should be pursued for linking laboratory and field studies: (1) field studies verifying the results of laboratory studies and vice versa; (2) studies of functional adjustment dealing with non-plasticity, which might be related to predicted and developmental adaptation as well as aging and dealing with plasticity related adaptive responses to many kinds of environmental stresses, which could be examined from the perspective of epigenetics; and (3) functional adjustment at the population level evaluated by identifying beneficial genotype variants for functional responses and the gene frequency and distributions. Other targets of study will of course be needed, but these three objectives should be prioritized. Linking laboratory and field studies will contribute to elucidating individual adaptability with reference to individual survival in terms of functional efficiency and tolerance to environmental stresses, as well as population adaptability with reference to the ability to leave offspring as evaluated by the frequency of gene-related beneficial functions with respect to the living environment.
